# Multifunctional mitoxantrone-conjugated magnetic nanosystem for targeted therapy of folate receptor-overexpressing malignant cells

**DOI:** 10.1186/s12951-015-0083-7

**Published:** 2015-03-26

**Authors:** Jaleh Barar, Vala Kafil, Mostafa Heidari Majd, Abolfazl Barzegari, Sajjad Khani, Mohammad Johari-Ahar, Davoud Asgari, George Cokous, Yadollah Omidi

**Affiliations:** Research Center for Pharmaceutical Nanotechnology, Tabriz, Iran; Faculty of Pharmacy, Tabriz University of Medical Sciences, Tabriz, Iran; Ludwig Centre for Cancer Research, University of Lausanne, Lausanne, Switzerland; Ovarian Cancer Research Centre, Perelman School of Medicine, University of Pennsylvania, Philadelphia, PA USA

**Keywords:** Magnetic nanoparticles, Target therapy, Folate receptor, Cancer, Apoptosis, Mitoxantrone, Nanomedicines, Theranostics

## Abstract

**Background:**

Targeted delivery of anticancer chemotherapeutics such as mitoxantrone (MTX) can significantly intensify their cytotoxic effects selectively in solid tumors such as breast cancer. In the current study, folic acid (FA)-armed and MTX-conjugated magnetic nanoparticles (MNPs) were engineered for targeted eradication of folate receptor (FR)-positive cancerous cells. Polyethylene glycol (PEG), FA and MTX were covalently conjugated onto the MNPs to engineer the PEGylated FA-MTX-MNPs. The internalization studies were performed using fluorescein isothiocyanate (FITC)-labeled FA-decorated MNPs (FA-FITC-MNPs) in both FR-positive MCF-7 cells and FR-negative A549 cells by means of fluorescence microscopy and flow cytometry. The cellular and molecular impacts of FA-MTX-MNPs were examined using trypan blue cell viability and FITC-labeled annexin V apoptosis assays and 4′,6-diamidino-2-phenylindole (DAPI) staining, DNA ladder and quantitative polymerase chain reaction (qPCR) assays.

**Results:**

The FR-positive MCF-7 cells showed significant internalization of the FA-FITC-MNPs, but not the FR-negative A549 cells. The FR-positive cells treated with the PEGylated FA-MTX-MNPs exhibited the IC_50_ values of 3 μg/mL and 1.7 μg/mL, 24 h and 48 h post-treatment, respectively. DAPI staining and DNA ladder assays revealed significant condensation of nucleus and fragmentation of genomic DNA in the FR-positive MCF-7 cells treated with the PEGylated FA-MTX-MNPs as compared to the FR-negative A549 cells. The FITC-labeled annexin V assay confirmed emergence of late apoptosis (>80%) in the FR-positive MCF-7 cells treated with the PEGylated FA-MTX-MNPs, but not in the FR-negative A549 cells. The qPCR analysis confirmed profound cytotoxic impacts via alterations of apoptosis-related genes induced by MTX-FA-MNPs in MCF-7 cells, but not in the A549 cells.

**Conclusion:**

Our findings evince that the engineered PEGylated FA-MTX-MNPs can be specifically taken up by the FR-positive malignant cells and effectively demolish them through up-regulation of Bcl-2–associated X protein (Bax) and Caspase 9 and down-regulation of AKt. Hence, the engineered nanosystem is proposed for simultaneous targeted imaging and therapy of various cancers overexpressing FRs.

## Introduction

Of different solid tumors, breast cancer is one of the most common life-threatening cancers among women. According to the World Health Organization (WHO) report on Dec. 2013, breast cancer incidences in the year 2012 has increased 20% as compared to the year 2008, resulting 521,000 death in 2012 [1].

Principally, breast cancer treatment modalities are based on surgery, radiotherapy, hormone-therapy and chemotherapy [2]. Of these treatment modalities, chemotherapy agents are used to induce cytotoxic impacts in cancerous cells through various mechanisms such as DNA detriments and inhibition of cell division and growth. For instance, MTX as one of the chemotherapeutic agents is commonly used for the treatment of metastatic breast cancer, acute myeloid leukemia and non-Hodgkin’s lymphoma. It hinders cell proliferation through inhibition of topoisomerase II and disruption of DNA repair/synthesis [3], intercalation of DNA [4], DNA damage and apoptosis via inhibition of the mitochondrial pathway [5]. Unfortunately, administration of MTX is associated with inevitable initiation of inadvertent side effects (e.g. weakness, hair loss, diarrhea, heart problems and immunosuppression), mainly because of the non-specific effects on the healthy cells/tissue [6,7]. Further, cancer cells may become resistant to MTX. To tackle such dilemmas, cancer cells must be targeted with smart drug delivery nanosystems (NSs) to deliver anticancer agents such as MTX specifically into the tumor microenvironment (TME) and hence malignant cells.

Among various NSs (e.g., nanoliposomes, polymeric NPs, dendrimers and other organic/inorganic NPs) designed for targeted therapy of cancer [8], MNPs appear to be one of the most promising delivery agents because they are biocompatible and can be easily decorated with homing and therapy agents [9]. Besides, potent toxic agents conjugated onto MNPs can be localized at the target site using an external magnetic field [10]. Targeted MNPs were shown to accumulate highly within the target tumor cells through passive and active targeting mechanisms while use of an external magnetic field can intensify the accumulation of MNPs [11-13]. Of various oncomarkers exploited for targeted therapy of cancer, folate receptors (FRs) have highly been overexpressed in various solid tumors such as breast and ovarian cancers [14,15]. Hence, FA, a safe small molecule also known as vitamin M or B9, has been used as homing device to target the FRs-overexpressing malignant cells. Owing to its versatility and conjugation simplicity, the FA-conjugation have been used for engineering multimodal nanomedicines and theranostics [16-19]. Surface modification of MNPs with polyethylene glycol (PEG) was shown to enhance the biocompatibility and the duration of blood circulation and to reduce the antigenicity of MNPs [20,21]. For example, Zhang *et al.* coated MNPs with PEG-FA and reported increased internalization of the modified MNPs in BT20 cells with decreased uptake in macrophages [22]. We have previously engineered multimodal PEGylated MNPs armed with FA and conjugated with MTX [23], or loaded with tamoxifen (TMX) [24]. We have also capitalized on functionalized MNPs to enhance the delivery of plasmid DNA into *Escherichia coli* [25]. In the current study, we aimed to study the cytotoxicity mechanism(s) of the PEGylated FA-MTX-MNPs in the FR-positive MCF-7 cells in comparison with the FR-negative A549 cells.

## Materials and methods

### Materials

Mitoxantrone was purchased from Ebewe Pharma GmbH (Unterach, Austria). Low melting point agarose, RPMI 1640 and fetal bovine serum were purchased from Invitrogen-Gibco (Paisley, UK). Ethylenediaminetetraacetic acid (EDTA), 4-(2-hydroxyethyl)-1-piperazineethanesulfonic acid (HEPES), streptomycin, penicillin G, L-glutamine, trypan blue solution (0.4%), fluorescein isothiocyanate dye (FITC), sodium dodecyl sulfate (SDS), propidium iodide (PI), sodium chloride (NaCl) and 4′, 6-Diamidino-2-phenylindole (DAPI) were purchased from Sigma-Aldrich (Poole, UK). Total RNA extraction RNeasy Mini Kit was purchased from Qiagen, Inc. (Valencia, CA, USA). Primers for real time PCR (*18srRNA*, *AKt, Caspase9,* and *Bax)* were purchased from Eurofins MWG Operon (Ebersberg, Germany). The SYBR® Green PCR master mix was obtained from Applied Biosystems (Foster City, USA). Murine leukemia virus reverse transcriptase (M-MLV), deoxynucleotide triphosphates (dNTPs), random hexamer (pdN6) and MgCl_2_ and other reagent not mentioned for RT-PCR were obtained from Fermentas (Crawley, UK). Annexin V-FITC apoptosis detection kit was obtained from EMD Chemicals (Gibbstown, NJ, USA). Cell culture dishes (well plates, pipette and flasks) were obtained from SPL Life Sciences (Pocheon, South Korea). MCF-7 and A549 cell lines were purchased from National Cell Bank of Iran, Pasteur Institute (Tehran, Iran).

### Engineering and morphological characterization

MNPs were synthesized, PEGylated and conjugated with FA and MTX as described previously [24]. The morphology and size of the engineered MNPs were characterized using transmission and scanning electron microscopies as reported previously [23,24].

### Particle size analysis

To determine the size of the engineered MNPs, we employed dynamic light scattering (DLS) using Nanotrac Wave™ (Microtrac, San Diego, CA, USA). The experiments were performed at room temperature. MNPs were specifically analyzed in terms of the hydrodynamic radius at a range of 0.8 to 6500 nanometers and zeta potential from −125 to +125 mV. The size of MNPs was calculated by fitting the data to a polydispersed model using the Dynamics software version 5.26 (Microtrac, San Diego, CA, USA).

### Atomic force microscopy (AFM) analyses

AFM analyses were performed on glass slides. Briefly, the glass slides were cleaned with acetone and washed with (3×) with Milli-Q deionized water, and dried under nitrogen flow. Then, 100 μL of the bare or modified MNPs were deposited on the glass slides. The slides were allowed to dry at room temperature. All AFM experiments were fulfilled by means of the contact mode using JPK AFM Nanowizard™ (JPK Instruments AG, Berlin, Germany) mounted on Olympus invert microscope IX81 (Olympus Corp., Tokyo, Japan). We used HYDRA2R-100NG silicon nitride cantilever (length 100 μm, width 35 μm and thickness 0.2 μm) with spring constant of 0.011 N/m and 15–29 kHz resonant frequencies (Applied Nano Structures Inc., Mountain View, CA, USA) containing silicon tip. All images were acquired in air at ambient condition with a scan rate of 1.2 Hz with I-gain, P-gain and set-point of 170 Hz, 0.0040 and 950 mV, respectively. The images were processed by Nanowizard Data Processing software version spm-4.2.62, and necessary adjustments were applied for the background slope, the contrast and brightness of images.

### Cell culture and treatments

MCF-7 and A549 cells were cultivated at a seeding density of 4 × 10^4^ cells/cm^2^ in 6-well plates using RPMI-1640 supplemented with 10% FBS, penicillin G (100 U/mL) and streptomycin (100 μg/mL) and maintained at 37°C in 5% CO^2^ and 95% air. At 50% confluence, the cultivated cells were exposed to a designated amount of free MTX, free MNPs or FA-MTX-MNPs (with 0.05, 0.2, 0.8, 1.6, 3.2 μg/mL of MTX) for 24 and 48 h. Then, the treated cells were subjected to cell viability assay.

### Trypan blue staining

Trypan blue staining was used for preliminary evaluation of cytotoxic effects of MTX alone, FA-MTX-MNPs and free MNPs in the FR-positive MCF-7 cells and the FR-negative A549 cells. After 24 h and 48 h treatment, the treated cells were exposed to 0.4% trypan blue and incubated for 5 min, and then analyzed by Olympus CKX41 light microscopy equipped with DP20 camera (Olympus Corp., Tokyo, Japan).

### Internalization assessment

To study the FR-mediated internalization of MNPs, we used MNPs decorated with FA and FITC (i.e., FA-FITC-MNPs). The cultivated cells in 6-well plates/cover slides were incubated with designated amount of FA-FITC-MNPs (~10.0 μg MNPs/mL) for 1 h at 37°C. After fixing with 4% paraformaldehyde, the cells were subjected to fluorescent microscopy analysis using Olympus IX81 invert fluorescence microscope equipped with Olympus DP70 camera (Olympus Corp., Tokyo, Japan) as described previously [23,24]. In parallel, for the quantitative revalidation, the FA-FITC-MNPs treated cells were analyzed by FACS-Calibur flow cytometry (Becton Dickinson, San Jose, USA). The FACs flow cytometry data were analyzed using freely available WinMDI software ver.2.9 (http://facs.scripps.edu/software.html).

### DNA ladder assay

To evaluate DNA fragmentation in the cells treated with FA-MTX-MNPs, DNA ladder assay was recruited using a standard protocol as reported previously [26]. Briefly, at 50% confluence, the cultivated cells treated with designated amount of MTX alone, free MNPs or FA-MTX-MNPs for 2 h. At certain time points, the treated cells were gently trypsinized and washed with phosphate buffered saline (PBS) by centrifugation at 250 × g for 10 min. The cells were then incubated with 0.1 mg/mL RNase A at 37°C for 1 h in lysate buffer [10 mmol/L Tris–HCl, 10 mmol/L EDTA, and 0.6% SDS (pH 7.5)]. After precipitation of protein contents with 5 mol/L NaCl by centrifugation at 10,000 × g for 60 min at 4°C, the DNA was purified from the supernatant using a standard chloroform-phenol extraction method. Isopropanol was added, and the mixture was stored overnight at −20°C. After centrifugation at 13,500 × g for 15 min at 4°C, the DNA pellet was re-suspended in Tris buffer [10 mmol/L Tris–HCl and 1 mmol/L EDTA (pH 8.0)]. The extracted DNA samples were then quantified by UV spectrophotometer at 260 nm and subjected to electrophoresis on 1.5% agarose gel and stained with ethidium bromide.

### Apoptosis detection by DAPI staining

The DAPI staining assay was conducted to detect possible occurrence of nucleus condensation in the treated cells. Briefly, the treated cells were fixed with the freshly prepared ice-cold paraformaldehyde (4%) and then exposed to 0.1% Triton X-100 in PBS for 5 min for permeabilization. They were subsequently stained with DAPI (1 μg/mL in PBS) for 5 min in the dark. After removing the surplus stain, the cells were washed (3×) using 0.1% Triton X-100 in PBS. The image acquisition was performed by Olympus IX81 invert fluorescence microscope equipped with Olympus DP70 camera (Olympus Corp., Tokyo, Japan) as described previously [27].

### Acridine orange – ethidium bromide (AO-EB) viability assessment

The AO-EB assay was conducted to further validate possible occurrence of early and late apoptosis and/or necrosis in the cells treated with MNPs (10.71 μg/mL), MTX (1.60 μg/mL) or FA-MTX-MNPs (12.31 μg/mL). Briefly, after washing (3×) with PBS, 40-50% confluent MCF-7 cells were treated with MNPs, MTX or FA-MTX-MNPs and then subjected to the staining with acridine orange (100 μg/mL) and ethidium bromide (100 μg/mL). The cells were then subjected to light/fluorescence microscopy analyses using Olympus IX81 invert fluorescence microscope equipped with Olympus DP70 camera (Olympus Corp., Tokyo, Japan).

### FITC-Labeled annexin V apoptosis assay

Annexin V staining was accomplished to detect any incidence of apoptosis using a protocol described previously [28]. Briefly, after washing (3×) with PBS, the treated cells were detached by tripsinization and 1.0 × 10^6^ cells were incubated with 100 μL of 1X binding buffer containing 5 μL FITC-labeled annexin V at 37°C in the dark for 10 min. The cells were then exposed to 200 μL PBS with 5 μL PI. After washing (3×) with PBS, the cells were subjected to the image acquisition using Olympus IX81 invert fluorescence microscope equipped with Olympus DP70 camera (Olympus Corp., Tokyo, Japan) as well as flow cytometry analysis using FACS-Calibur flow cytometr (Becton Dickinson, San Jose, USA).

### Quantitative real-time PCR

For the real time PCR analysis, total RNA was extracted from treated cells using Qiagen RNeasy® plus mini Kit (Qiagen GmbH, Hilden, Germany) following the manufacturer protocol. Possible genomic DNA contamination was eliminated from the extracted total RNA by treating the mixture with 1 U/μL of RNase-free DNase at 37°C for 30 min. Afterword, the enzyme was heat inactivated using 5 mM EDTA (10 μL) at 65°C for 5 min. The quality and quantity of the DNase treated total RNAs were determined by NanoDrop 1000 (NanoDrop, Wilmington, USA). For the synthesis of cDNA, RT reaction was performed using 1 μg of total RNA, 0.5 μL RNase inhibitor (40 U/μL), 10 μL 10X RT buffer, 0.5 μL random hexamer (400 ng/μL), 2 μL dNTPs mix (10 μM) and 1 μL RT enzyme (200 U/μL) in a total volume of 50 μL. The RT cycling program was as follows: primary denaturation at 95°C for 5 min, incubation at 25°C for 10 min, 42°C for 42 min and 95°C for 5 min. The real time PCR was fulfilled using Bio-Rad iQ5 system (Bio-Rad Laboratories Inc., Hercules, USA) using the following thermal cycling conditions: an initial denaturation step at 95°C for 10 min, and 40 cycles of 95°C for 15 sec, annealing temperature for 1 min (62°C for 18srRNA and Caspase 9, 53°C for Bax and 58.3°C for AKt), and extension at 72°C for 30 sec. Each reaction was performed in the final volume of 25 μL, containing 12.5 μL 2X master mixes, 1 μL cDNA, and 1 μL from each primer (100 nM) which was designed by Beacon Designer 7 (primers are listed in Table 1). All reactions were independently performed in triplicates. RNAse/DNAse free water (1 μL) and extracted RNA (1 μL) without DNase treatments were used as negative and no template controls, respectively. The 18srRNA were used as a housekeeping gene for the normalization of CT values as described previously [29].

**Table 1:** **Primers used for amplification of selected genes**

**Gene**	**Primer sequence**	**Melting T (C°)**
*18srRNA*	Forward: 5′-CGATGCGGCGGCGTTATTC-3′(19)	62
NR_003286.1	Reverse:5′-TCTGTCAATCCTGTCCGTGTCC-3′(22)	
*Akt*	Forward: 5′- CGCAGTGCCAGCTGATGAAG -3′(20)	58.3
NM_005163.2	Reverse: 5′- GTCCATCTCCTCCTCCTCCTG -3′(21)	
*Caspase 9*	Forward: 5′- TGCTGCGTGGTGGTCATTCTC-3′(21)	62
NM_001229.2	Reverse: 5′- CCGACACAGGGCATCCATCTG-3′(21)	
*Bax*	Forward: 5′- GATGCGTCCACCAAGAAG -3′(18)	53
NR_027882	Reverse: 5′- AGTTGAAGTTGCCGTCAG-3′(18)	

### Statistical analysis

All data obtained from PCR and cell viability analyses were exhibited as mean ± standard deviation (SD). Statistical assessments of data were performed using one-way analysis of variance (ANOVA) and/or Student’s *t*-test with a p value less than 0.05 for statistical significance.

## Results

### Synthesis and characterization

**Figure 1:**
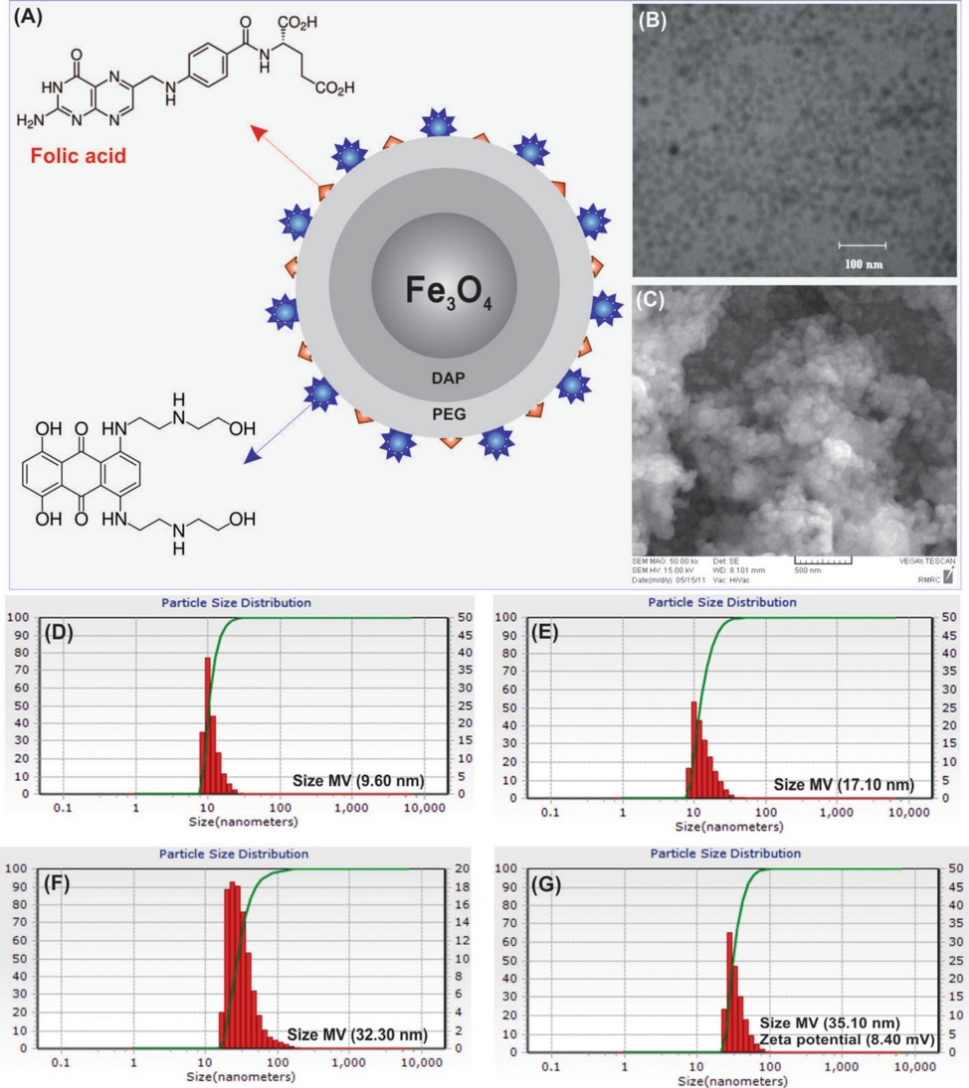
**Architecture, morphology, size and zeta potential of the engineered MNPs. A)** PEGylated FA-armed MTX-conjugated MNPs (not drawn to scale). **B)** TEM micrograph of PEGylated FA-MTX-MNPs. **C)** SEM micrograph of PEGylated FA-MTX-MNPs. **D)** DLS image of bare MNPs. **E)** DLS image of Fe_3_O_4_-DPA-PEG NPs. **F)** DLS image of Fe_3_O_4_-DPA-PEG-FA NPs. **G)** DLS image of PEGylated FA-MTX-MNPs. FA: folic acid. MTX: mitoxantrone. MNPs: magnetic nanoparticles. TEM: transmission electron microscopy. SEM: scanning electron microscopy. DLS: dynamic light scattering.

Figure 1 (panel A) shows schematic representation of MNPs. Iron oxide (Fe_3_O_4_), the core of NPs, was prepared through the thermal decomposition reaction of Fe(acac)_3_ method. The MNPs (~7-10 nm) was modified by dopamine-polyethylene glycol-folic acid (DPA-PEG-FA), in which the bromoacetyl (BrAc) terminal polyethylene glycol dopamine (DPA-PEG-BrAc) was synthesized and treated with ethylene diamine to form bifunctional PEG moiety containing dopamine at one end and amino group at the other end (i.e., DPA-PEG-NH_2_). It was then reacted with MNPs to form Fe_3_O_4_-DPA-PEG-NH_2_ NPs. The activated FA was covalently coupled to Fe_3_O_4_-DPA-PEG-NH_2_ NPs forming Fe_3_O_4_-DPA-PEG-FA. MTX molecules were then conjugated to Fe_3_O_4_-DPA-PEG-FA to form PEGylated FA-MTX-MNPs (Figure 1A).

The bare and functionalized MNPs were analyzed by TEM and SEM (Figure 1, panels B and C, respectively) as well as DLS and AFM. Based on DLS analyses, the size of bare MNPs, Fe_3_O_4_-DPA-PEG NPs, Fe_3_O_4_-DPA-PEG-FA NPs and PEGylated FA-MTX-MNPs were respectively 7–10, 17–20, 30–32 and 33–35 nm (Figure 1, panels D, E, F and G, respectively). The PEGylated FA-MTX-MNPs displayed zeta potential value of 8–10 mV.

**Figure 2:**
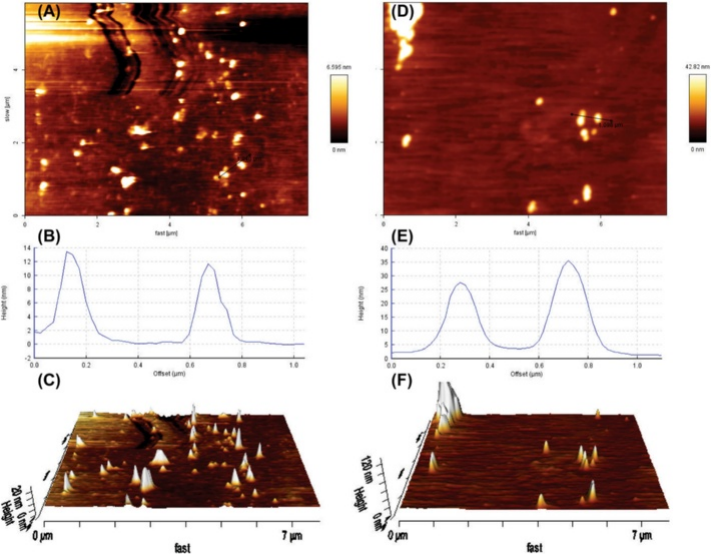
**AFM analyses of the bare MNPs and the PEGylated FA-MTX-MNPs.** AFM analyses were performed using intermittent contact mode. Panels **A**, **B** and **C** respectively represent the height (trace), the cross section line profile, and the 3D images of the bare MNPs. Panels **D**, **E** and **F** respectively represent the height (trace), the cross section line profile, and the 3D images of the PEGylated FA-MTX-MNPs. AFM: atomic force microscopy. FA: folic acid. MTX: mitoxantrone. MNPs: magnetic nanoparticles.

AFM analyses confirmed the results obtained by DLS analyses showing the size (height) of ~10 and ~35 nm for the bare MNPs and the PEGylated FA-MTX-MNPs, respectively (Figure 2).

### Cellular uptake and internalization

**Figure 3:**
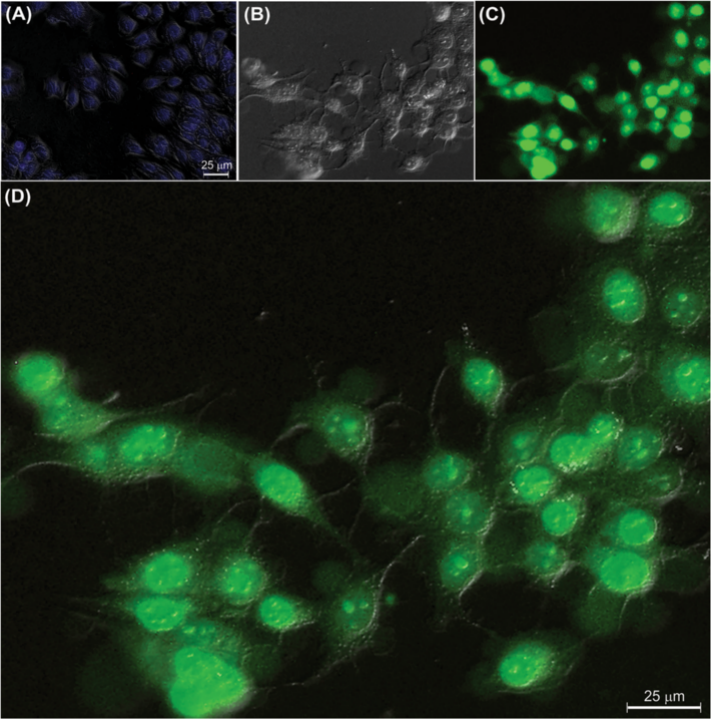
**The internalization of PEGylated FA-FITC-MNPs in A549 and MCF-7 cells.** The cultivated cells were treated with the FA-FITC-MNPs for 2 h and then subjected to the light and fluorescence microscopy analyses. **A)** DIC and FM superimposed image of the A549 cells. **B)** DIC, **C)** FM, and **D)** DIC and FM superimposed images of the MCF-7 cells. FA: folic acid. FITC: Fluorescein isothiocyanate. MNPs: magnetic nanoparticles. DIC: differential interference contrast. FM: fluorescence microscopy.

**Figure 4:**
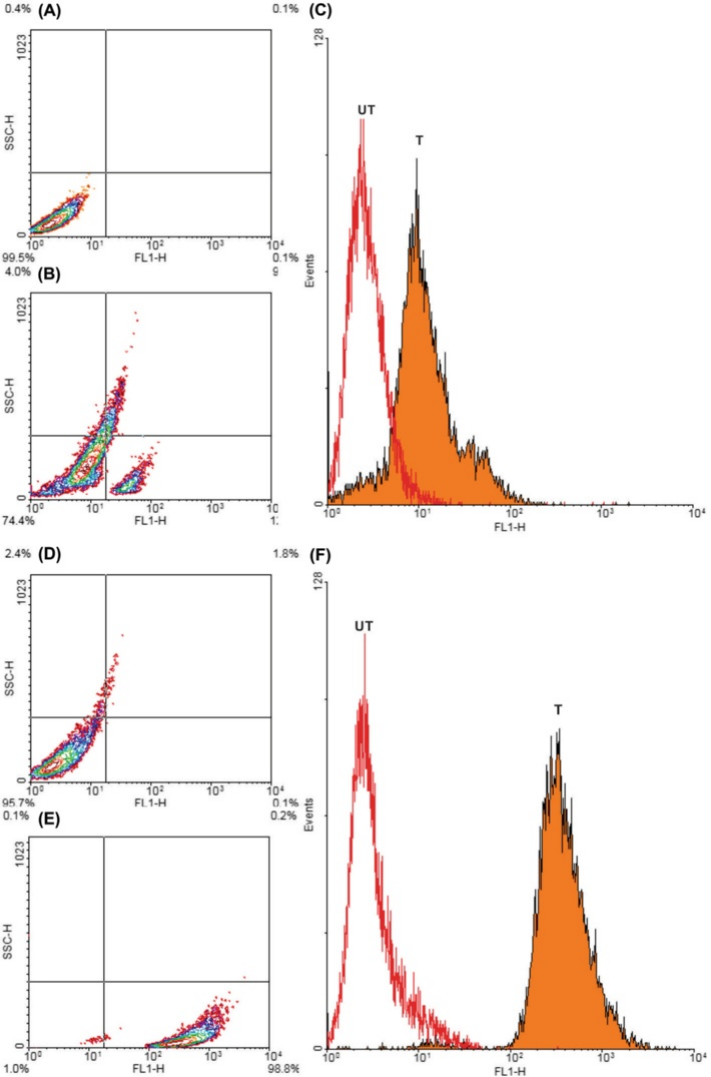
**Flow cytometry analyses of A549 and MCF-7 cells treated with the PEGylated FA-FITC-MNPs.** The cultivated cells were treated with the FA-FITC-MNPs for 2 h and then subjected to the flow cytometry analyses. Panels **A**, **B** and **C** show the untreated, the treated and the overlaid images of the A549 cells, respectively. Panels **D**, **E** and **F** represent the untreated, the treated and the overlaid images of the MCF-7 cells, respectively. FA: folic acid. FITC: Fluorescein isothiocyanate. MNPs: magnetic nanoparticles.

In vitro cellular internalization was examined in both MCF-7 and A549 cells. As shown in Figure 3, FA-armed FITC-conjugated MNPs were significantly taken up by the FR-positive MCF-7cells, but not the FR-negative A549 cells. The flow cytometry was used to revalidate the fluorescence microscopy. As shown in Figure 4, the flow cytometry analysis confirmed the results obtained by the fluorescence microscopy analysis, showing markedly high internalization of the engineered PEGylated FA-MTX-MNPs by the FR-positive MCF-7 cells (>70%). However, trivial internalization of these NSs was observed by the FR-negative A549 cells.

### Trypan blue exclusion assay

**Figure 5:**
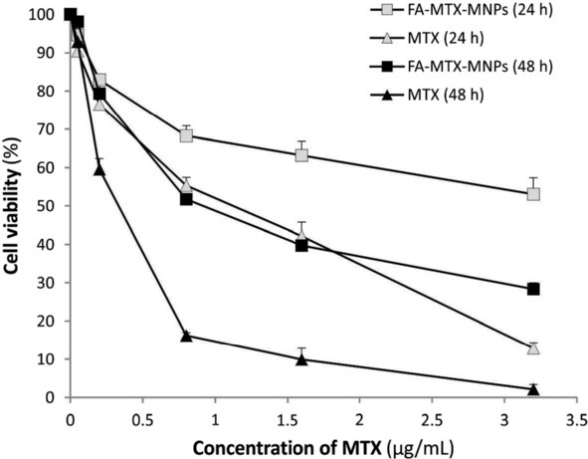
**Cell viability trypan blue exclusion analysis in MCF-7 cells.** The cultivated cells at 50% confluency were exposed to either the free MTX (0.05-3.20 μg/mL) or the PEGylated FA-MTX-MNPs (0.39-24.62 μg/mL) for 24 h and 48 h, and then subjected to the trypan blue exclusion assay. FA: folic acid. MTX: mitoxantrone. MNPs: magnetic nanoparticles.

To study the toxicity of FA-MTX-MNPs, trypan blue exclusion assay was used. As shown in Figure 5, the PEGylated FA-MTX-MNPs were able to significantly repress the growth and the proliferation of MCF-7cells (p < 0.01), but not A549 cells (data not shown). The IC_50_ for the free MTX and the PEGylated FA-MTX-MNPs were respectively 1.5 μg/mL and 3.0 μg/mL after 24 h, and 0.86 μg/mL and 1.7 μg/mL after 48 h.

### DNA fragmentation analysis

**Figure 6:**
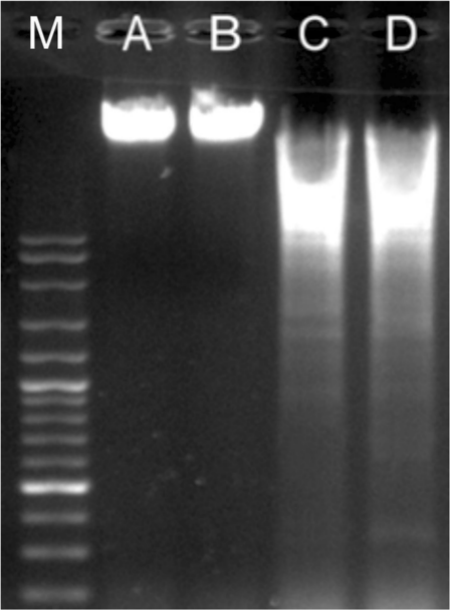
**DNA ladder assay for detection of DNA damages in MCF-7 cells.** Lanes A, B, C and D respectively represent the untreated control cells, the treated cells with the free MNPs (10.71 μg/mL), the PEGylated FA-MTX-MNPs (12.31 μg/mL), or the free MTX (1.60 μg/mL) treated cells for 48 h. M: Marker (100 base-pair). FA: folic acid. MTX: mitoxantrone. MNPs: magnetic nanoparticles.

The most important feature of apoptotic cells is the cleavage of chromosomal DNA at the internucleosomal sites into numerous units including 180–200 base pairs fragments. The engineered PEGylated FA-MTX-MNPs impacts on the integrity of genomic DNA was examined. Figure 6 epitomizes a typical DNA fragmentation within the MCF-7 cells treated with the PEGylated FA-MTX-MNPs.

### DAPI staining

**Figure 7:**
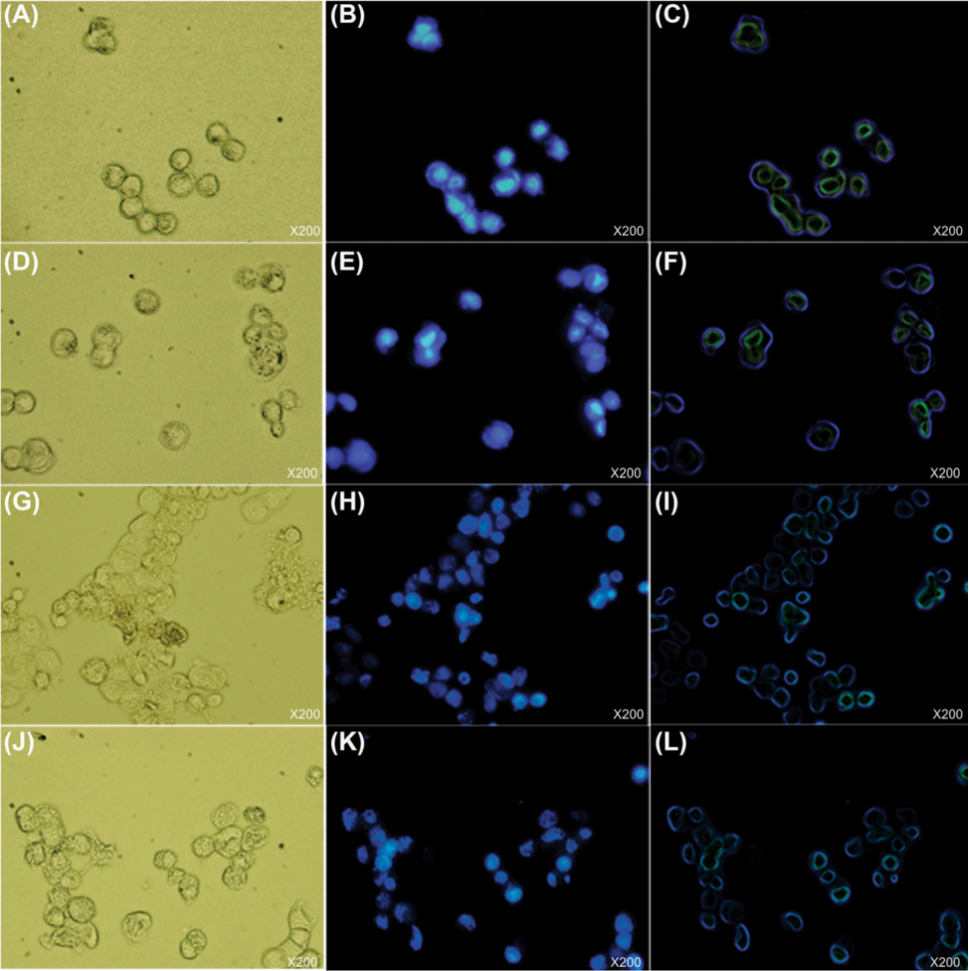
**DAPI staining assay for detection of chromatin decomposition in MCF-7 cells.** Cell were treated with either the free MTX (1.60 μg/mL) or the PEGylated FA-MTX-MNPs (12.31 μg/mL), stained with DAPI and then subjected to the transmission light microscopy (panels **A**, **D**, **G** and **J**), fluorescence microscopy (panels **B**, **E**, **H** and **K**) and the stylized images of fluorescence microscopy (panels **C**, **F**, I and **L**) showing margins of nucleuses. All images are shown as 200× magnification. Panels A, B and C show the untreated control cells. Panels **D**, **E** and **F** represent the cells treated with the free MNPs alone. Panels **G**, **H** and **I** show the cells treated with the free MTX. Panels **J**, **K** and **L** demonstrate the cells treated with the PEGylated FA-MTX-MNPs.

Nuclear fragmentation and chromatin condensation/remodeling that are typical markers of apoptosis were evaluated in the MCF-7 cells treated with the free MTX, the free MNP or the PEGylated FA-MTX-MNPs using DAPI staining assay. As shown in Figure 7, the FA-MTX-MNPs imposed clear changes in the nucleus (most likely through DNA condensation and chromatin alteration) of the treated MCF-7 cells, while the free MTX treated cells did not show similar effects (Figure 7). The responses of A549 cells were negligible as compared to that of MCF-7 cells.

### AO-EB viability analysis

**Figure 8:**
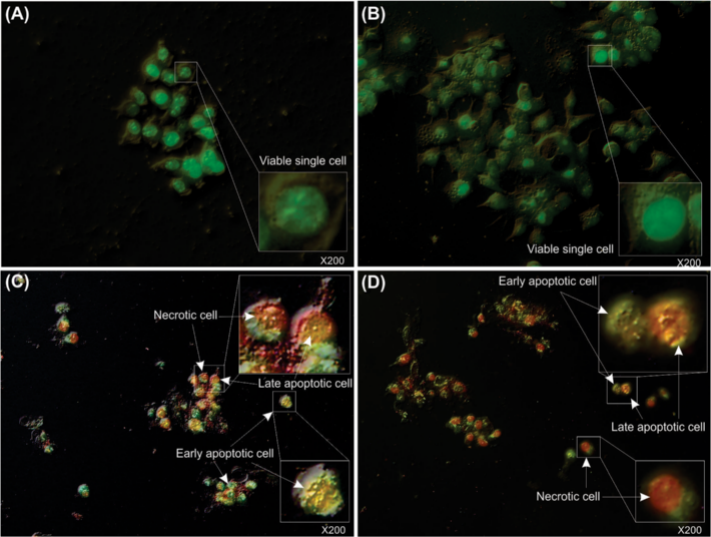
**Acridine orange – ethidium bromide (AO – EB) assay for detection of apoptotic and/or necrotic MCF-7 cells. A)** Untreated cells as a negative control. **B)** Cells treated with the free MNPs (10.71 μg/mL). **C)** Cells treated with the free MTX (1.60 μg/mL). **D)** Cells treated with the PEGylated FA-MTX-MNPs (12.31 μg/mL). The cultured cells, at 40-50% confluency, were treated and then subjected to the staining with acridine orange (100 μg/mL) and ethidium bromide (100 μg/mL) followed by light/fluorescence microscopy analyses. The untreated cells (panel **A**) and the MNPs treated cells (panel **B**) were viable with impermeable membrane to EB, which showed normal round nuclei stained green. The cells treated with the free MTX or the PEGylated FA-MTX-MNPs (panels **C** and **D**, respectively) were apoptotic (stained orange) with condensed and/or fragmented nuclei or necrotic (stained red).

To detect the early and late apoptosis as well as necrosis upon treatment of the MCF-7 cells treated with the free MNPs, the free MTX molecules or the FA-MTX-MNPs, the cells were stained with AO (100 μg/mL) and EB (100 μg/mL) and analyzed by the light/fluorescence microscopy. Figure 8 represents the AO-EB stained MCF-7 cells. Having possessed integrated membrane, the untreated viable cells were impermeable to EB and hence they displayed normal round nuclei stained green (Figure 8A). In the same way, the MNPs treated cells were found to be viable (Figure 8B). The cells treated with either the free MTX (Figure 8C) or the FA-MTX-MNPs (Figure 8D) showed profound apoptosis and/or necrosis. The apoptotic cells had condensed and/or fragmented nuclei (Figure 8C and D). They appeared to be impermeable to EB during the early stages of apoptosis showing nuclei stained green, but permeable to EB during the later stages of apoptosis showing nuclei stained red. The necrotic cells displayed red nuclear stain with no nuclear condensation.

### Annexin V apoptosis assay

**Figure 9:**
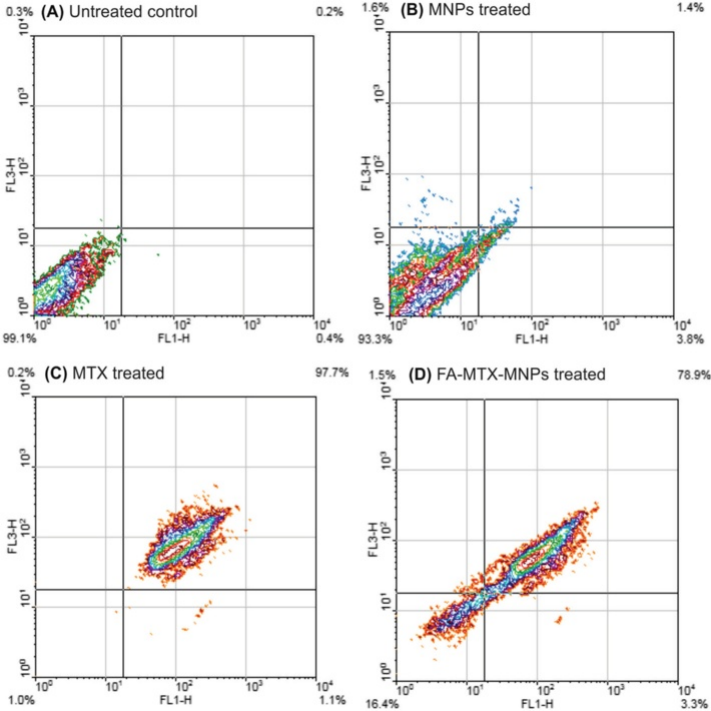
**FITC-conjugated annexin V apoptosis analysis of MCF-7 cells. A)** The untreated cells (negative control). **B)** The cells treated with the free MNPs (10.71 μg/mL). **C)** The cells treated with the free MTX (1.60 μg/mL). **D)** The cells treated with the PEGylated FA-MTX-MNPs (12.31 μg/mL). FITC: Fluorescein isothiocyanate. FA: folic acid. MTX: mitoxantrone. MNPs: magnetic nanoparticles.

FITC-annexin V apoptosis assay was employed to explore the apoptosis stage in the treated cells with the PEGylated FA-MTX-MNPs. Technically, phosphatidylserine (Ptd-L-Ser) is mostly located on the cytosolic leaflet of cell membranes in mammalian cells while it is transmitted to the outer plasma membrane leaflet when apoptosis is initiated. It is a susceptible marker for the occurrence of early phase of apoptosis [30], in which the annexin V shows high affinity to the negatively charged Ptd-L-Ser and binds to the target molecule in the presence of Ca^2+^ [31]. As analyzed by the fluorescence microscopy, there exists a significant FITC^+^/PI^+^ of cells treated with the PEGylated FA-MTX-MNPs or the free MTX molecules. As shown in Figure 9, the flow cytometry analyses revealed occurrence of the late phase apoptosis in more than 95% of the MCF-7 cells, but not the A549 cells, upon treatment with the PEGylated FA-MTX-MNPs.

### Quantitative gene expression

**Figure 10:**
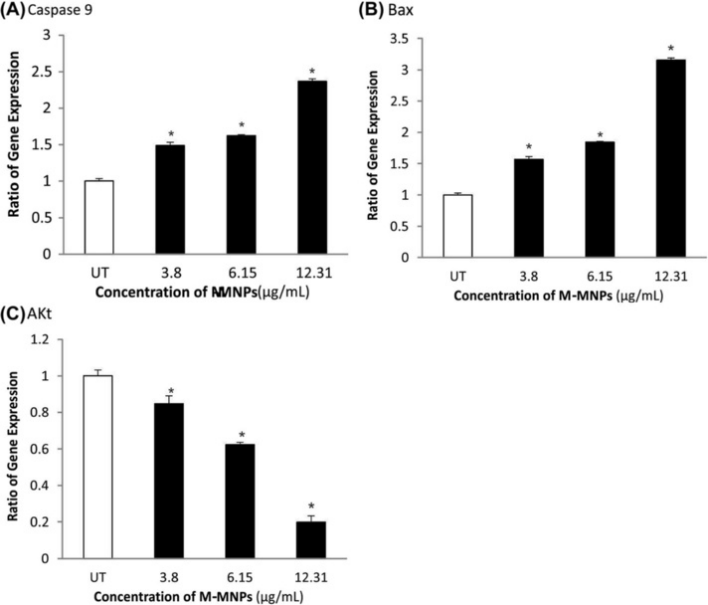
**Quantitative PCR analysis of apoptotic genes in MCF7 cells treated with the PEGylated FA-MTX-MNPs.** Panels **A**, **B**, and **C** show the expression of Caspase 9, BAx and AKt, respectively. As a housekeeping gene, 18 s rRNA was used to normalize Ct values using the Pfaffl method. Data are expressed as mean values of independent triplicates (mean ± SD). Asterisks represent statistical difference (p < 0.05) with untreated control. FA: folic acid. MTX: mitoxantrone. MNPs: magnetic nanoparticles. Ct: threshold cycle. UT: untreated control.

To find the mechanism of the apoptosis, quantitative gene expression was performed looking at three key genes involved in apoptosis and cell proliferation (i.e., Bax, AKt and Caspase 9). Figure 10 demonstrates the expression changes of Bax, AKt and Caspase 9 in responses to designated amounts of the PEGylated FA-MTX-MNPs in a concentration-dependent manner. Increased amount of the PEGylated FA-MTX-MNPs appeared to significantly increase the expression of Bax (P = 0.009) and Caspase 9 (P = 0.011), while the expression of AKt (P = 0.002) was found to be decreased.

## Discussion

Multifunctional nanomedicines are deemed to revolutionize the treatment of life-threatening diseases such as malignancies. While carrying therapeutic agents, these NSs can be armed with homing and imaging devices which enable them to be used for specific simultaneous targeting and imaging. Of various advanced nanomaterials such as quantum dots and carbon nanotubes [32,33], MNPs provide great characteristics for surface functionalization. We have previously reported on engineering of various NSs for effective delivery of different drugs [34-40], and also showed that the surface functionalized MNPs can further be conjugated with anticancer drugs such as MTX [23], loaded with TMX [24], or even self-assembled with plasmid DNA [25]. In the current study, we aimed to study the specific targeting, internalization and genomic impacts of the engineered multifunctional MNPs in the FR-positive/negative cells. The TEM, DLS (Figure 1) and AFM (Figure 2) analyses revealed the size of the engineered MNPs to be approximately about 10 and 35 nm before and after modification. The PEGylated FA-MTX-MNPs showed surface charge of 10 mV. The size and zeta potential characteristics of these NSs make them to be freely distributed with no aggregations. It is noteworthy to mention that small NPs (<10 nm) can be quickly removed from the blood circulation through the clearance functions of kidney and/or liver, while the larger particles show tendency to be cleared by mononuclear phagocyte system, the so-called reticuloendothelial system (RES) [41]. Of note, different size ranges of PEGylated NPs and also non-PEGylated NPs (with size range around 100 nm) have a longer circulation duration and lower kidney/hepatic filtration [42]. Furthermore, the effect of NPs size (ranging from 10 nm to 100 nm) was studied [43]. It was found that the penetration of NPs into the tumors can extremely be dependent on the size of the NPs. It can be deduced that the smaller the NPs, the higher the rate of the accumulation of NPs within the tumor. Hence, we speculate that the engineered PEGylated FA-MTX-MNPs with a size range at ~35 nm might show better penetration into solid tumors through receptor-mediated endocytosis via folate receptor, which was confirmed by our preliminary studies. Given the fact that the tumor vasculature is irregularly un-integrated and the pressure of interstitial fluid in solid tumors is significantly higher than the normal tissues [44,45], the engineered FA-MTX-MNPs are speculated to be able to extendedly circulate within the blood and effectively accumulate into the tumor interstitium and hence cancer cells. In addition, the morphological and physicochemical characterization of the PEGylated FA-MTX-MNPs by TEM, SEM and AFM revealed regular spherical shape without any obvious detriments in consensus with previous reports [46]. Taken into consideration that many synthetic non-biodegradable polymers and lipids used for delivery of drugs/genes are able to elicit inadvertent toxicogenomics leading to inevitable undesired cellular responses [47-54], we speculate the PEGylated FA-MTX-MNPs to be a safer delivery NS with no intrinsic nonspecific biological impacts. Similar effects have also been reported for the shikonin-loaded antibody-armed poly(lactic acid-co-glycolic acid) NPs for targeted therapy of ovarian cancer [34].

Based upon greater internalization of the PEGylated FA-MTX-MNPs, it can be pondered that they may provide robust means for ligand-target delivery of chemotherapies. Some previously published studies have shown the internalization mechanisms of various NSs through endocytosis machineries in different cells [55,56]. It seems that the internalization of folate-decorated NPs is a size-dependent phenomena medicated via either clathrin-coated pits or caveolae-mediated endocytosis [57]. For example, NPs with size range about 50 nm appear to internalize quickly selectively through the clathrin-mediated endocytosis, while the internalization of NPs with size range at ~250 nm seems to be a slow process via the caveolae-mediated endocytosis as a dominant path. Internalization of MNPs was reported to be via the clathrin-coated pits in LNCaP prostate cancer cells [58], which can also be augmented by an external magnetic field [59].

Furthermore, FRs have been shown to be overexpressed by various epithelial cancerous cells in different solid tumors, and hence they can be exploited for the targeted therapy of cancer [60-65]. Accordingly, targeting cancer cells overexpressing FRs by NPs armed with FR-specific homing devices have been shown to result in profound internalization of the NPs [23,25,66]. In our study, the fluorescence microscopy examinations (Figure 3) and the flow cytometry analyses (Figure 4) revealed that the FA-armed MNPs decorated with FITC were actively taken up by the FR-positive MCF-7 cells, but not the FR-negative A549 cells. It should be stated that MNPs fascinated much interests not only due to their magnetic characteristics but also because of their association with low toxicity in the human body [67-69]. For example, Karlsson *et al.* compared cytotoxicity of various MNPs and inferred that the MNPs do not induce cytotoxicity at a concentration range under 100 μg/mL. Our study disclosed that, while the MNPs per se were nontoxic, the PEGylated FA-MTX-MNPs could considerably induce high inhibitory impacts on the proliferation of FR-positive cancer cells as compared to the untreated control cells (Figure 5). Such inhibition appeared to be a concentration-dependent phenomenon. The liberation of covalently conjugated MTX payloads from the engineered MNPs was shown to be a sustained-release process, in which the esterase-mediated enzymatic activity of the cancer cells are responsible for the release of drug molecules [70], reader is directed to see the following work for some selected methods of surface modifications and bioconjugations of NPs [35]. The free MTX molecules induce the inhibitory impacts via interaction with DNA and inhibition of topoisomerase II enzyme by ensnaring it with a covalent topoisomerase-DNA complex [71]. Besides, the inhibition in DNA helicase II activity has also been shown as a mechanism for DNA damages induced by MTX [72]. The MTX molecules can result in profound cell death, in which the biosigns for such cell death appear to manifest as the chromatin condensation/remodeling [73], and the fragmentation in the internucleosomal DNA [74]. Previous studies have also highlighted an enhanced fragmentation of internucleosomal DNA by the anthracyclines drugs such as MTX in the human myeloid leukemia HL-60 and KG-1 cells [75]. In the current study, both the free MTX and the PEGylated FA-MTX-MNPs were found to induce the fragmentation of internucleosomal DNA in the MCF-7 cells, which was confirmed by DNA ladder assay (Figure 6). In addition, the fluorescence microscopy examination of DAPI stained cells illustrated chromatin rings and crescent-shaped nuclei within the nuclear membrane of the cells treated with the free MTX and the PEGylated FA-MTX-MNPs (Figure 7). Given that the PEGylated FA-MTX-MNPs elicit the cell death, we assumed that the impacts of these NSs might be as same as that of the free MTX molecules in MCF-7 cells. However, to ensure upon impacts of the PEGylated FA-MTX-MNPs, we also studied the alternation of membrane phospholipids using annexin V flow cytometry apoptosis assay based on the existence of Ptd-L-Ser on the plasma membrane as a sign of annexin V affinity for the apoptotic cells [76]. Once the nuclear morphology and internucleosomal DNA fragmentation of the treated cells were characterized, we found somewhat DNA fragmentation (Figure 6) and chromatin condensation (Figure 7) in MCF-7 cells upon treatment with the free MTX or the PEGylated FA-MTX-MNPs. Of note, we also witnessed high degrees of phenotypic apoptosis and necrosis within MCF-7 cells treated with either the free MTX or the engineered PEGylated FA-MTX-MNPs, while the free MNPs per se appeared to be safe as shown by the AO-EB apoptosis/necrosis detection assay (Figure 8). In fact, the AO is a vital dye that can stain both live and dead cells, but the EB stains solely the cells with defected membrane. While the viable cells appeared to be uniformly green (Figure 8A and B), the early apoptotic cells stained green with bright green dots in the nuclei evincing the chromatin condensation and the nuclear fragmentation (Figure 8C and D). Late apoptotic cells appeared to incorporate the EB and hence stained orange showing condensed and often fragmented nuclei, but the necrotic cells stained orange/red showing almost normal nuclear morphology without any condensed chromatin (Figure 8C and D).

Further, the MCF-7 cells treated with the PEGylated FA-MTX-MNPs revealed a high affinity to the annexin V, showing the expression of Ptd-L-Ser on the outer membrane leaflet. The annexin V assay revealed that approximately 80% of the cells treated with FA-MTX-MNPs were FITC^+^/PI^+^, indicating their ability to induce Ptd-L-Ser on the outer membrane leaflet of the treated cells and to loss the membrane integrity which inhibits PI exclusion by the cells (Figure 9). We speculate that the engineered PEGylated FA-MTX-MNPs have great ability to initiate the “find-me” and “eat-me” signals on the surface of the cells, and hence activate the cell death mechanism in a similar fashion as reported for the free MTX molecules previoulsy [77].

To delineate the apoptosis pathway observed in the FR-positive MCF-7 cells treated with the PEGylated FA-MTX-MNPs, we studied the gene expression profile of several essential genes related to the mitochondrial apoptosis pathway. It was found that the cell death signals are generated through the formation of pores in the membrane leading to liberation of mitochondrial proteins like small mitochondria-derived activator of caspases (SMACs) into the cytoplasmic matrix. Further, it has already been reported that the interaction of some released factors with the apoptotic protease activating factor-1 (Apaf-1) known as apoptosome can activate the pro-caspase 9 [78]. The consequently activated caspase 9 can in turn cleave the subsequent proteins in the apoptotic caspase cascade, which can irretrievably oblige the cells to assign an inevitable intrinsic apoptosis. The PI3K/AKt pathway, as an important intracellular signaling path, plays a key role in apoptosis and subsequently in cancer development [79], in particular breast cancer. Through this pathway, phosphorylation of the inactive form of AKt can inhibit Bax effects on the mitochondria, in large part by entrapping the protein in the cytosol [80,81], resulting in inevitable immoderate proliferation of cancerous cells. However, upon the initiation of apoptosis via PI3K/AKt pathway, Bax interacts with the outer mitochondrial membrane voltage-dependent anion channels (VDAC) and hence increases the permeability of the mitochondrial membrane [82]. Opening of the mitochondrial channels can lead to the release of cytochrome complex (Cyt c) and other pro-apoptotic proteins of the mitochondria, resulting in profound stimulation of the mitochondrial apoptotic pathway. In our investigation, we studied the expression of the most important genes (i.e., AKt, Caspase 9 and Bax) and observed a considerable regulation in the gene expression profile of the treated cells with the PEGylated FA-MTX-MNPs (Figure 10). The expression of AKt was down-regulated as compared to the untreated control cells. Nonetheless, there was a significant up-regulation in the expression of AKt’s downstream gene (i.e. Bax), which was not surprising because of the down-regulation of AKt. Taken all, the use of PEGylated FA-MTX-MNPs can activate the tumor suppressor genes Bax and Caspase 9, but inactivate the tumor inducer gene AKt, which triggers the apoptosis pathway(s). Thus, given these changes in expression AKt, Caspase 9 and Bax, we presume that the initiation of the observed apoptosis by the engineered MNPs may occur via the PI3K/AKT pathway that is known as the main pathway involved in breast cancer.

## Conclusion

So far, a number of studies have been conducted towards development of effective anti-cancer nanomedicines in order to lower the inadvertent adverse effects of chemotherapeutics. In the current study, we developed FA-armed MNPs conjugated with MTX to specifically target the FR-positive MCF-7 cells and effectively deliver the MTX molecules to the target cells. We studied the effects of surface modification on cellular uptake, toxicity and mechanism(s) of the toxicity of the engineered PEGylated FA-MTX-MNPs. Our results revealed that the FA-coated MNPs have a considerably higher level of cellular uptake by the FR-positive MCF-7 cells but not the FR-negative A549 cells. The observed growth inhibitory effects induced by the engineered NSs in the FR-positive MCF-7 appeared to be dose- and time-dependent, which were comparable to the overall biological impacts of the free MTX molecules. Based on the profound inhibitory effects of the PEGylated FA-MTX-MNPs in the FR-positive cancer cells through PI3K/AKT pathway, we propose this FR-targeting NS as a robust site-specific targeted delivery nanomedicine and theranostic that can be used against various FR-positive malignancies. Having capitalized on passive targeting through enhanced permeability and retention effect and active targeting through folate receptor-mediated endocytosis we expect to attain greater specific endocytic internalization and hence better clinical outcome by these nano-sized PEGylated FA-MTX-MNPs.

## Abbreviations

MTX: Mitoxantrone
FA: Folic acid
MNPs: Magnetic nanoparticles
FA-MTX-MNPs: Folic acid armed and mitoxantrone conjugated magnetic nanoparticles
FR: Folate receptor
qPCR: Quantitative polymerase chain reaction
DAPI: 4′,6-diamidino-2-phenylindole
FITC: Fluorescein isothiocyanate
Bax: Bcl-2–associated X protein
WHO: World Health Organization
NSs: Nanosystems
TME: Tumor microenvironment
NPs: Nanoparticles
PEG: Polyethylene glycol
TMX: Tamoxifen
TEM: Transmission electron microscopy
SEM: Scanning electron microscopy
FACS: Fluorescence activated cell sorting
EDTA: Ethylenediaminetetraacetic acid
HEPES: 4-(2-hydroxyethyl)-1-piperazineethanesulfonic acid
PI: Propidium iodide
M-MLV: Murine leukemia virus reverse transcriptase
dNTPs: Deoxynucleotide triphosphates
pdN6: Random hexamer
SDS: Sodium dodecyl sulfate
NaCl: Sodium chloride
PBS: Phosphate buffered saline
