# Erratum to: Multifunctional mitoxantrone-conjugated magnetic nanosystem for targeted therapy of folate receptor-overexpressing malignant cells

**DOI:** 10.1186/s12951-015-0108-2

**Published:** 2015-09-25

**Authors:** Jaleh Barar, Vala Kafil, Mostafa Heidari Majd, Abolfazl Barzegari, Sajjad Khani, Mohammad Johari-Ahar, Davoud Asgari, George Coukos, Yadollah Omidi

**Affiliations:** Research Center for Pharmaceutical Nanotechnology, Tabriz, Iran; Faculty of Pharmacy, Tabriz University of Medical Sciences, Tabriz, Iran; Ludwig Centre for Cancer Research, University of Lausanne, Lausanne, Switzerland; Ovarian Cancer Research Centre, Perelman School of Medicine, University of Pennsylvania, Philadelphia, PA USA

## Erratum to: Journal of Nanobiotechnology (2015) 13:26 DOI 10.1186/s12951-015-0083-7

In the original publication of this work [1], the surname of author George Coukos was misspelt as George Cokous.
